# The Effect of Exercise Training During Pregnancy to Improve Maternal Vascular Health: Focus on Gestational Hypertensive Disorders

**DOI:** 10.3389/fphys.2020.00450

**Published:** 2020-05-08

**Authors:** Isabel Witvrouwen, Dominique Mannaerts, An M. Van Berendoncks, Yves Jacquemyn, Emeline M. Van Craenenbroeck

**Affiliations:** ^1^Research Group Cardiovascular Diseases, Department of Genetics, Pharmacology and Physiopathology of Heart, Blood Vessels and Skeleton, University of Antwerp, Antwerp, Belgium; ^2^Department of Cardiology, Antwerp University Hospital, Antwerp, Belgium; ^3^Antwerp Surgical Training, Anatomy and Research Centre, University of Antwerp, Antwerp, Belgium; ^4^Department of Obstetrics and Gynaecology, Antwerp University Hospital, Antwerp, Belgium

**Keywords:** exercise, pregnancy, vascular adaptation, pre-eclampsia, gestational hypertension

## Abstract

Hypertensive disorders of pregnancy, including gestational hypertension and pre-eclampsia, occur in up to 10% of pregnancies and are associated with increased life-long cardiovascular risk. Physical activity improves cardiovascular health in pregnancy and may lower the risk of developing hypertensive disorders of pregnancy. However, a minority of pregnant women comply with the recommended level of physical activity. Adequate knowledge on the physiological effects of exercise in healthy pregnancy could help to overcome potential barriers as pregnancy is a unique window of opportunity to improve health outcomes for both mother and child. In this mini review, we discuss structural and functional vascular adaptations during healthy and hypertensive pregnancies, we elaborate on the effects of exercise on the vasculature and review the safety and existing evidence of exercise training as preventive therapy for gestational hypertensive disorders.

## Introduction

Worldwide guidelines recommend aerobic training during pregnancy from 60 to 150 min/week (Savvaki et al., 2018). Little is known about the number of women practicing this, but numbers as low as 15% have been cited (Kuhrt et al., 2015). Women who exercise as recommended have 30% less risk for developing gestational hypertensive disorders (GHD), including gestational hypertension (GH), characterized by hypertension initiating after the 20th pregnancy week and pre-eclampsia (PE) defined as hypertension and proteinuria after the 20th pregnancy week (Magro-Malosso et al., 2017; Davenport et al., 2018b). Preliminary data suggest that exercise during pregnancy has a lifelong protective effect resulting in a reduced cardiovascular risk profile in the perimenopause (Clapp, 2008). Maternal physical exercise is also beneficial for the fetus, resulting in less macrosomia and consequently improved cardiovascular health of the child at a later age (Alexander et al., 2015).

Pregnancy can be considered a stress test for the cardiovascular system, imposing profound cardiovascular adaptations including increased blood volume, accompanied by a drop in vascular resistance due to increased angiogenesis and vasodilation, generalized reduction in arterial stiffness and improved endothelial function, increased cardiac output associated with increased right and left chamber size and eccentric hypertrophy, resulting in higher stroke volume and heart rate and a fall in systemic blood pressure (Melchiorre et al., 2012; Chung and Leinwand, 2014; Osol and Bernstein, 2014; Tkachenko et al., 2014; Mannaerts et al., 2019). Regular physical exercise can boost these adaptations as has been demonstrated for angiogenesis and endothelial function (Skow et al., 2017). In women with GHD, these functional and structural vascular adaptions fail (Mannaerts et al., 2019), and may persist beyond pregnancy (Kirollos et al., 2019), explaining why these women are at a lifelong increased risk for cardiovascular disease (Lane-Cordova et al., 2019).

In this mini review, we will elucidate the vascular adaptation during normal vs. hypertensive pregnancies and we will focus on the potentially beneficial effects of exercise on the vasculature. Based on this concept, physical exercise prior to and during pregnancy may be a promising therapy to prevent GHD and GHD recurrence, however, current data to underscore this hypothesis are still limited.

## Vascular Adaptation During Healthy Pregnancy

An optimal adaptation of the cardiovascular system is crucial for a healthy pregnancy. As early as 5 weeks amenorrhea, a significant fall in systemic vascular tone occurs, altering the set-points of the baroreceptors and the stretch receptors (Tkachenko et al., 2014). As a result, systemic vascular resistance decreases to allow sufficient placental perfusion (Clark et al., 1989). Venous tone decreases as well, resulting in expansion of the venous compartment and increased cardiac preload, ultimately leading to increased cardiac output (Melchiorre et al., 2012; Chung and Leinwand, 2014). To accommodate this blood volume expansion and increased cardiac output, the arterial bed needs to undergo structural and functional changes (Skow et al., 2017).

During pregnancy, **structural arterial remodeling** is mainly driven by placental growth factor (PlGF)-induced angiogenesis, occurring primarily at the uteroplacental unit (Osol and Bernstein, 2014). Soluble fms-like tyrosine kinase 1 (sFlt-1) is the circulating form of the VEGF receptor-1 and binds VEGF and PlGF thereby reducing their bioavailability.

The ratio of sFlt-1/PlGF is an important indicator of the angiogenic status in pregnancy and is used to predict and diagnose PE. Interestingly, this ratio appears to be indicative of future vascular dysfunction risk (Zeisler et al., 2016). The decrease in total vascular resistance is mediated by VEGF and PlGF as they induce distal angiogenesis (Hasan et al., 2002). Placental growth factor also mediates the cardiac adaptation and insufficient PlGF leads to impaired ventricular remodeling and cardiac dysfunction (Hochholzer et al., 2011).

To accommodate the increased blood volume while maintaining low blood pressure, a generalized reduction in **arterial stiffness** is of great importance. Central (aortic) pulse wave velocity (PWV), the gold standard for arterial stiffness, is known to be decreased in healthy pregnancy (Mannaerts et al., 2019).

A healthy **endothelium** controls vasomotor tone, which is essential during pregnancy. The rapidly expanding blood volume and increase in cardiac output pose an increased shear stress on endothelial cells, resulting in increased endothelial nitric oxide (NO) production (Cockell and Poston, 1997; Williams et al., 1997). Together with higher estrogen levels, this leads to a systemic vasodilation (Meah et al., 2016). In healthy pregnancy, endothelial NO synthase (eNOS) activity is significantly increased (Nelson et al., 2000) which is mirrored in improved flow-mediated dilatation (FMD), the gold standard for endothelial function measurement (Iacobaeus et al., 2017; Mannaerts et al., 2019).

## Vascular Maladaptation in Gestational Hypertensive Disorders

Women who develop hypertensive disorders during pregnancy such as GH or PE appear to fail the stress test of pregnancy, in part due to insufficient cardiovascular adaptation. Therefore, the risk of developing cardiovascular disease later in life is 9.5 times higher for women with severe early PE [hazard ratio (HR) = 9.5, 95% confidence interval (CI) = 4.5–20.3] (Mongraw-Chaffin et al., 2010). Furthermore, PE has been associated with an increased risk for developing end-stage kidney disease (HR = 4.96, 95% CI = 3.9–6.3) (Khashan et al., 2019). Therefore, long-term cardiovascular monitoring and early preventive therapy are advocated (McDonald et al., 2008; Ahmed et al., 2014).

**FIGURE 1:**
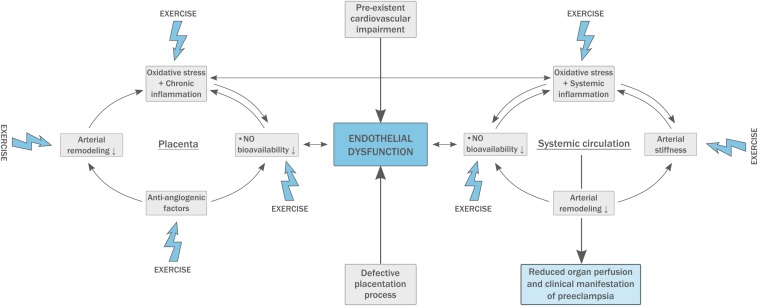
Pathophysiology of pre-eclampsia (PE). A pre-existing fragile endothelial situation leads to defective placentation and high circulating levels of oxidative stress. This inadequate response to pregnancy results in arterial stiffness and exacerbates generalized endothelial dysfunction. Physical exercise has beneficial effects on multiple components of the model.

In PE, insufficient **arterial remodeling** at the spiral arteries results in placental ischemia-reperfusion damage and the production of high amounts of free radicals causing oxidative stress (Figure 1). Circulating free radicals activate peripheral leucocytes and platelets, resulting in an inflammatory state and disturbing proper endothelial function. The reaction of oxidative products with NO decreases its bioavailability which impairs endothelial function even more (Mannaerts et al., 2018). The abundant placental ischemia and oxidative stress in PE results in an anti-angiogenic state with a three-fold increase in antiangiogenic factors (sFlt-1) and a 90% reduction in angiogenic factors (PlGF and VEGF; Tomimatsu et al., 2017).

Women suffering from PE have increased **arterial stiffness** both during and after pregnancy, and arterial stiffness is directly correlated to the severity of the disease (Figure 1; Hausvater et al., 2012; Mannaerts et al., 2019). Carotid-femoral PWV is abnormal from 11 to 13 weeks in patients who develop PE later in pregnancy, which supports the concept that PE is not caused by dysfunctional placentation alone and underlying vascular disease must be present. Increased arterial stiffness may have an important influence on fetal birth weight and pregnancy outcome (Skow et al., 2017). In addition, central PWV is strongly related to an increased risk for the development of cardiovascular disease later in life, also in PE (Hausvater et al., 2012).

PE is characterized by dysfunction of both resting (L-FMC, low-flow mediated constriction) and recruitable (FMD) endothelial capacity (Mannaerts et al., 2019). **Endothelial dysfunction** is proven to be present prior to the development of PE, possibly serving as a predictive parameter (Figure 1; Weissgerber, 2014). Further, women with a history of PE appear to have reduced FMD up to 3 years postpartum (Scholten et al., 2014). Endothelial dysfunction impairs vascular smooth muscle relaxation which enhances arterial stiffness and plays an important role in the development of atherosclerosis. This suggests endothelial dysfunction to be the most plausible common link between the pathophysiology of PE and future cardiovascular disease (Mosca et al., 2011; Weissgerber, 2014).

## Effects of Exercise on the Vasculature

**FIGURE 2:**
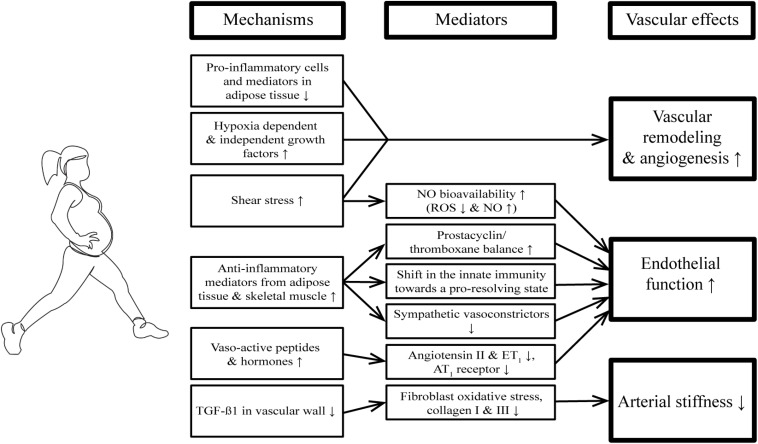
Beneficial effects of repeated exercise bouts on the vasculature. AT1 receptor, Angiotensin II receptor type 1; eNOS, endothelial nitric oxide synthase; ET1, endothelin-1; NO, nitric oxide; ROS, reactive oxygen species; TGF-β, transforming growth factor beta.

Repeated exercise bouts effectively benefit vascular function directly by exerting shear forces on the vascular wall (Hambrecht et al., 2003; Adams et al., 2005; Grimm et al., 2018) and indirectly by the release of anti-inflammatory and anabolic mediators in response to increased muscular energy demands (Goldhammer et al., 2005; Kadoglou et al., 2007; Pedersen et al., 2007; Rehm et al., 2015). This results in functional adaptation of the local and systemic vasculature to meet increased perfusion demands and to structural arterial remodeling by engagement of neuro-humoral and metabolic mechanisms (Figure 2; Roveda et al., 2003; Adams et al., 2005; Pedersen et al., 2007; Rehm et al., 2015).

There is clear evidence that **endothelial function** is improved by regular physical activity, both in patients with cardiovascular risk factors (Lavrenčič et al., 2000) and in patients with established cardiovascular disease (Linke et al., 2001; Van Craenenbroeck et al., 2010; Van Craenenbroeck E.M. et al., 2015). This exercise-induced benefit on endothelial function is mediated by different factors.

First, increased shear stress during exercise activates eNOS and reduces NAD(P)H oxidase activity, resulting in decreased reactive oxygen species (ROS) and increased NO bioavailability (Hambrecht et al., 2003; Adams et al., 2005). Furthermore, laminar shear stress prevents inflammation-related alterations in eNOS levels and prostacyclin/thromboxane ratio in an atherogenic environment (Grimm et al., 2018).

Second, endurance training has repeatedly been reported to lower levels of pro-inflammatory cytokines (CRP, IL-18, IL-1β, and IL-8) and increase anti-inflammatory cytokines (IL-10; Goldhammer et al., 2005; Kadoglou et al., 2007). The reduction of body fat and the anti-inflammatory and anabolic mediators released from the active skeletal muscle (referred to as “myokines”; Pedersen et al., 2007) induce systemic shifts in the innate and adaptive immunity toward a more pro-resolving and anti-inflammatory status (Rehm et al., 2015).

Third, exercise training modulates the balance between vasodilating and vasoconstricting factors, overall resulting in more vasodilation. Exercise training reduces levels of endothelin-1 and noradrenalin (Mortensen et al., 2009; Dow et al., 2017), reverses the aging-induced increase in the vasoconstrictor thromboxane (Hellsten et al., 2012) and lowers sympathetic tone (Roveda et al., 2003).

Regular physical activity and exercise interventions have been associated with the prevention of age-related increases in **arterial stiffness** (Fleenor et al., 2010). In a mouse model, the profibrotic cytokine TGF-β1 increased with aging in the carotid adventitia, where it augmented oxidative stress in fibroblasts. This resulted in increased collagen I and III deposition, and arterial stiffness (Fleenor et al., 2010). The aging-associated elevation in adventitial TGF-β1 is reduced by aerobic exercise both in mice and humans, which in turn reduces large elastic artery stiffening (Fleenor et al., 2010). In addition, increased oxidative stress has been associated with reduced large elastic artery compliance in sedentary vs. habitually exercising postmenopausal women (Moreau et al., 2006).

Exercise has a profound impact on the process of **vascular remodeling**, which is again driven by increased blood flow and shear stress, by inflammatory cells, as well as by hypoxia-dependent and -independent growth factors (Hoier and Hellsten, 2014; Laughlin, 2016). The pro-angiogenic effect of exercise is not limited to the exercising skeletal muscle, but also induces angiogenesis in adipose tissue (Van Pelt et al., 2017) and increased coronary collateral flow in patients with coronary artery disease (Möbius-Winkler et al., 2016).

## Effects of Exercise in Healthy Pregnancy

Regular exercise is known to decrease cardiovascular disease in the non-pregnant population and is implemented in the treatment of heart failure and coronary artery disease patients (Karlsen et al., 2019; Witvrouwen et al., 2019a). Improved vascular health has been suggested as a major contributing factor (Myers, 2003; Van Craenenbroeck E.M. et al. 2010; 2015, Van Craenenbroeck A.H. et al., 2016).

In an uncomplicated pregnancy, current guidelines recommend moderate exercise at a frequency of two to four times a week and with an exercise duration of 30 min, throughout pregnancy (Savvaki et al., 2018). Overall, both aerobic and resistance exercises do not exert any adverse effects during pregnancy. However, evidence on resistance training is scarce and exercise with heavy loads is discommended (Savvaki et al., 2018). Most recreational exercise is safe, but sports that may cause abdominal trauma, falls or excessive joint stress and scuba diving should be avoided (Kuhrt et al., 2015; Bø et al., 2016; Savvaki et al., 2018).

Whereas the guidelines generally recommend 30 min of moderate exercise two to four times per week, 85% of pregnant women are exercising below these levels (Evenson and Wen, 2010). The most frequent barriers are fatigue, lack of time and pregnancy discomforts, but also safety concerns such as low birth weight, preterm labor and inducing fetal bradycardia could withhold pregnant women and health practitioners to prescribe the recommended amount of physical exercise (Kuhrt et al., 2015; Coll et al., 2017; Harrison et al., 2018; Witvrouwen et al., 2019b). Adequate knowledge on the physiological effects of exercise training in healthy pregnancy should help to overcome these barriers as pregnancy is a unique window of opportunity to improve health outcomes for the mother and also the future generations (Kuhrt et al., 2015).

There is no evidence for the induction of preterm delivery by regular physical activity. On the contrary, even a reduction in preterm birth of 20–50% in women performing exercise during pregnancy compared with sedentary pregnant women has been shown (Juhl et al., 2010).

The same is true for the concerns regarding exercise and low birth weight: maternal exercise was not associated with low birth weight or Apgar score at delivery (Davenport et al., 2018a). The normalization of maternal blood glucose, decrease in insulin resistance and increased placental functional capacity and nutrient delivery are suggested mechanisms to explain the beneficial effect of exercise on birth weight (Clapp, 2003; Kuhrt et al., 2015).

During exercise, peripheral vasodilation in the skin and exercising muscles can lead to reduced placental blood flow. In addition to poor autoregulation of the placental circulation, this may cause reduced oxygen and nutrient delivery to the fetus. Other proposed mechanisms for possible fetal distress during maternal exercise include vagal reflex, cord compression or fetal head compression related to malposition (Artal and O’Toole, 2003). Nevertheless, a significant decrease in mean uterine artery blood flow and fetal bradycardia has only been shown in Olympic level athletes exercising at more than 90% of the maximal maternal heart rate (Salvesen et al., 2011). Moreover, it has been shown that regular exercise improves both maternal cardiovascular adaptations and placental function to maintain sufficient fetal oxygenation and growth and does not adversely affect fetal heart rate (Clapp, 2003; Kuhrt et al., 2015).

## Effects of Exercise for the Prevention of Hypertensive Disorders of Pregnancy

*Even prior to actual pregnancy, physical activity* is related to a lower occurrence of PE, with a 22–35% relative risk (RR) reduction for women with the highest vs. lowest physical activity level (Aune et al., 2014). This risk was even further reduced (RR = 0.64, 95% CI = 0.44–0.93) with combined pre- and early pregnancy physical activity. When assessing the dose-response effect of physical activity, 5–6 h of physical activity per week reduced the risk of PE with 40%, but no further reduction with increasing activity levels were reported (Aune et al., 2014). Likewise, sedentary behavior has been related to higher odds for the development of PE and GH (Aune et al., 2014; Fazzi et al., 2017; Davenport et al., 2018b).

Whether *physical activity and training during pregnancy can prevent GH and PE*, remains to be established. The largest systematic review and meta-analysis to date on GH (22 randomized controlled trials (RCTs), *n* = 5,316) and PE (15 RCTs, *n* = 3,322) showed that exercise during pregnancy significantly lowered the risk for GH (OR = 0.61, 95% CI = 0.43–0.85) and PE (OR = 0.59, 95% CI = 0.37–0.94). Moreover, 600 MET-min/week of moderate-intensity exercise (the equivalent of 140 min of brisk walking) was accompanied by a 25% reduction in the odds of developing GH, PE and gestational diabetes mellitus, with a clear dose-dependent effect (Davenport et al., 2018b).

This is in line with findings from three other large meta-analyses, where reductions in PE or GHD were observed (Aune et al., 2014; Di Mascio et al., 2016; Magro-Malosso et al., 2017). However, other systematic reviews and meta-analyses reported conflicting results depending on the type of the study-design (cohort studies vs. case-control studies vs. RCTs) and the exercise exposure that was studied (Kasawara et al., 2012; Wolf et al., 2013; Muktabhant et al., 2015; da Silva et al., 2016; Zheng et al., 2017; Table 1).

**TABLE 1:** Summary of meta-analyses and systematic reviews on the effect of exercise before and/or during pregnancy and the occurrence of gestational hypertensive disorders.

**References**	**No. studies, No. participants included**	**Exercise exposure**	**Risk reduction (95% confidence interval)**
(Davenport et al., 2018b)	(1) GH: 32 RCTs; *n* = 9,648	(1) Exercise with/without cointerventions vs. no exercise during pregnancy (pooled estimate)	(1) GH: OR = 0.81, 95% CI = 0.65–1.0
Meta-analysis	(2) GH: 22 RCTs; *n* = 5,316	(2) Exercise-only interventions vs. no exercise during pregnancy (sensitivity analysis)	(2) GH: OR = 0.61, 95% CI = 0.43–0.85
	(3) PE: 26 RCTs; *n* = 10,177	(3) Exercise with/without cointerventions vs. no exercise during pregnancy (pooled estimate)	(3) PE: OR = 0.89, 95% CI = 0.73–1.08
	(4) PE: 15 RCTs; *n* = 3,322	(4) Exercise-only interventions vs. no exercise during pregnancy (sensitivity analysis)	(4) PE: OR = 0.59, 95% CI = 0.37–0.94

(Aune et al., 2014)	(1) Seven cohort and four case-control studies; *n* = 168,602	(1) High vs. low early pregnancy physical activity	(1) PE: RR = 0.79, 95% CI = 0.70–0.91
Meta-analysis	(2) Two case-control and 1 cohort study; *n* = 5,194	(2) High- vs. low-intensity activity in early pregnancy	(2) PE: RR = 0.51, 95% CI = 0.37–0.71
	(3) Four cohort and one case-control study; *n* = 10,317	(3) High vs. low prepregnancy physical activity	(3) PE: RR = 0.65, 95% CI = 0.47–0.89
	(4) One case-control and one cohort study; *n* = 4,240	(4) High- vs. low-intensity prepregnancy physical activity	(4) PE: RR = 0.55, 95% CI = 0.25–1.21
	(5) One cohort and two case-control studies; *n* = 5,291	(5) Combined physical activity before and during early pregnancy vs. no physical activity	(5) PE: RR = 0.89, 95% CI = 0.59–1.35

(Di Mascio et al., 2016) Meta-analysis	Nine RCTs; *n* = 2,059	35–90 min of aerobic exercise for 3–4 times per weeks vs. no exercise, randomized before 23 weeks	PE and GH: RR = 0.21, 95% CI = 0.09–0.45

(Magro-Malosso et al., 2017)	(1) Seven RCTs; *n* = 2,517	30–60 min of aerobic exercise for 2–7 times/week vs. no exercise, randomized before 23w	(1) GHD: RR = 0.70, 95% CI = 0.53–0.93
Meta-analysis	(2) Sixteen RCTs; *n* = 4,641		(2) GH: RR = 0.54, 95% CI = 0.40–0.74
	(3) Six RCTs; *n* = 2,230		(3) PE: RR = 0.79, 95% CI = 0.45–1.38

(Kasawara et al., 2012)	(1) Six case-control studies; *n* = 9,929	(1) LTPA, occupational activities and planned physical exercise vs. no physical activity	(1) PE: OR = 0.77, 95% CI = 0.64–0.91
Systematic review	(2) Ten cohort studies; *n* = 184,243	(2) LTPA, occupational activities and planned physical exercise vs. no physical activity	(1) PE: OR = 0.99, 95% CI = 0.93–1.05
	(3) One RCT; *n* = 79 (Yeo et al., 2008)	(3) Stretching vs. walking exercise 5 times per week from week 18 until the end of pregnancy	(3) PE: OR = 6.34, 95% CI = 0.72–55.37

(Wolf et al.,2013) Systematic review	Four case-control (*n* = 4,867) and 7 cohort studies (*n* = 166,822)	LTPA before and/or during pregnancy	(1) Light- or moderate-intensity LTPA: no association with PE.
			(2) Vigorous-intensity LTPA before and/or during pregnancy may reduce the risk of PE.
			(3) Reduced risk among women who participated in LTPA at least 25 times/month or > 4h per week
			(4) Elevated risk of severe PE with high amounts of LTPA, defined as ≥ 4.5 h per week

(da Silva et al., 2016) Meta-analysis	(1) Three RCTs; *n* = 1,417	LTPA in pregnancy vs. no physical activity	(1) PE: RR = 0.93, 95% CI = 0.55–1.57
	(2) Eight cohort studies; *n* = 155,414		(2) Similar findings; no evidence of an association between LTPA in pregnancy and PE

(Zheng et al., 2017) Meta-analysis	Five RCTs; *n* = 1,872	Exercise during pregnancy vs. usual daily activities	PE (secondary outcome): OR = 1.05, 95% CI = 0.53–2.07

(Muktabhant et al., 2015)	(1) Eight RCTs; *n* = 3,139	(1) Diet and exercise vs. standard care	(1) PE: RR = 0.99 95% CI = 0.74–1.31
Systematic review	(2) Three RCTs; *n* = 1,024	(2) Supervised exercise vs. standard care	(2) PE: RR = 0.91, 95% CI = 0.52–1.60
	(3) Two RCTs; *n* = 229	(3) Unsupervised exercise vs. standard care	(3) PE: RR = 1.60, 96% CI = 0.38–6.73

*GH, gestational hypertension; GHD, gestational hypertensive disorders; LTPA, leisure time physical activity; OR, odds ratio; PE, preeclampsia; RCT, randomized controlled trial; RR, relative risk.*

This controversy may be caused by methodological issues, such as heterogeneity in study designs or training programs. There is a wide variety in exercise type (strength vs. endurance vs. combined strength and endurance training, or stretching exercises), duration and frequencies of the training programs (with differences in number of sessions per week, the duration of these sessions and the total duration of the training intervention) in the current studies, and also the exercise domain (such as leisure time physical activity, occupational, domestic, or active commuting exercise) often differs. Furthermore, different evaluation of physical activity (objective measures such as accelerometry or subjective self-reported questionnaires), inadequate correction for confounding variables (some studies did not take BMI into account), or low training adherence could contribute to this discrepancy. The slightly stronger association between prepregnancy exercise and PE compared with early pregnancy physical activity, could also be due to higher achievable intensity levels before pregnancy compared with the pregnant state (Aune et al., 2014).

Conceptually, exercise in early pregnancy can reduce the risk of PE by ameliorating placentation since repetitive hypoxia bouts and reduced placental perfusion will stimulate cell proliferation and angiogenesis and lead to an improved sFlt-1/PlGF balance (Skow et al., 2017).

In elite athletes, evidence on a positive effect of vigorous exercise during pregnancy on PE or GH is lacking. A J-shaped relationship between the risk of PE and exercise, with a 40% reduction in risk with up to 5–6 h exercise per week, but no further reductions at higher activity levels has been described (Aune et al., 2014). As stated above, fetal adverse effects have only been shown in athletes exercising at more than 90% of the maximal maternal heart rate (Salvesen et al., 2011). Therefore, pregnant athletes should be referred to gynecologists for individual risk-assessment and recommendations regarding the type and intensity of exercise during pregnancy (Sma Position Statement et al., 2016; Mottola et al., 2018).

To date, only two RCTs evaluated the *effect of exercise on the recurrence of PE in a subsequent pregnancy* (Yeo et al., 2008; Kasawara et al., 2013). In the study of Kasawara et al., one training session per week in trimester 2 and 3 of pregnancy did not prevent PE recurrence. The low training intensity (heart rate 20% above resting value) and frequency demand for cautious interpretation of these results (Kasawara et al., 2013). Yeo et al. studied the effect of walking vs. stretching (5 × 40 min/week) in 79 women and also did not demonstrate a reduction in the incidence, possibly affected by low adherence (Yeo et al., 2008).

In established PE pregnancies, *only one RCT assessed whether exercise* (supervised stretching vs. autogenic training) *reduced blood pressure*. In 40 PE pregnancies, both training modalities equally lowered blood pressure and proteinuria (*p* < 0.05) over time (Awad et al., 2019).

## Current Research GAPS and Future Directions

A large body of evidence demonstrates that exercise improves systemic endothelial function and arterial stiffness in a wide range of subjects, from children to elderly, as well as in several diseases. Surprisingly, effects of exercise on the vasculature in healthy pregnancies is understudied and data in PE pregnancies are virtually non-existent. To our knowledge, only one study examined the effect of exercise training during a healthy pregnancy on endothelial function (Ramírez-Vélez et al., 2011). In that study, FMD improved by 30% by exercise training starting between 16 and 20 weeks, at moderate intensity. Concerning arterial stiffness, a discretely improved PWV in early post-partum period was observed with prenatal exercise, but has not been studied during pregnancy (Kawabata et al., 2012). In women with a history of PE, improved FMD and venous compliance with exercise training have been shown in small patient groups (Krabbendam et al., 2009; Scholten et al., 2014, 2015), and requires confirmation in larger trials.

Whether exercise training can prevent subsequent GHD in high risk patients, is a justified research question that deserves a well-designed clinical trial. Future research should focus on strategies to improve adherence to exercise training during pregnancy (supervised vs. unsupervised training, providing information on training characteristics and safety of exercise, etc.). Also, clear definitions of exercise should be used, using the FITT acronym (frequency, intensity, type, and time). These training characteristics should be compared and their effects on vascular health and the recurrence of GHD should be assessed. The role of gestational weight gain and the socioeconomic state of the women should be explored. Furthermore, confounding variables (age, BMI, parity, and smoking) and pre-pregnancy physical activity levels should be taken into account. Physical activity should be assessed using preferably objective measures. Also, more research on the timing of initiation of exercise (first, second, or third trimester of pregnancy) and more exercise-only interventions in overweight or obese women should be performed. In addition, whether post-partum exercise in women with history of PE can reduce their increased cardiovascular risk, deserves attention.

In the meantime, physical activity in pregnant women should be stimulated, with structured advice from the treating physician. Offering eg. a smartphone-based program while considering the socioeconomic and psychological needs should ultimately lead to fitter pregnant women, with clear benefits for mother and child.

## Conclusion

In GHD, structural and functional adaptations of the vascular wall fail by a large amount, leading to measurable effects on blood pressure in the acute phase and increased cardiovascular risk of both mother and child in the long term. Regular physical activity has profound effects on several parts of the vascular wall by improving endothelial function, reducing arterial stiffness and inducing angiogenesis. Nevertheless, whether these beneficial vascular effects of exercise are related to the lower risk on GHD following training remains to be confirmed. However, moderate physical exercise during pregnancy is safe and will benefit both short- and long-term outcome of mother and baby. Therefore, physical activity should be encouraged in every healthy woman considering only a few contra-indications and addressing potential barriers for exercise during pregnancy.

## Author Contributions

YJ wrote the introduction. DM wrote the part on vascular adaptation in healthy pregnancy and in hypertensive disorders of pregnancy. EV elaborated on the effects of exercise on the vasculature and edited the manuscript. AV described the effects of exercise in healthy pregnancy. IW discussed the effects of exercise for the prevention of hypertensive disorders of pregnancy and edited the manuscript. DM, IW, and EV wrote the current research gaps and future directions. All authors revised and accepted the final version of the manuscript to be published.

## Conflict of Interest

The authors declare that the research was conducted in the absence of any commercial or financial relationships that could be construed as a potential conflict of interest.
